# Prior Specification for More Stable Bayesian Estimation of Multilevel Latent Variable Models in Small Samples: A Comparative Investigation of Two Different Approaches

**DOI:** 10.3389/fpsyg.2020.611267

**Published:** 2021-01-25

**Authors:** Steffen Zitzmann, Christoph Helm, Martin Hecht

**Affiliations:** ^1^Hector Research Institute of Education Sciences and Psychology, University of Tübingen, Tübingen, Germany; ^2^Institute for the Management and Economics of Education, University of Teacher Education Zug, Zug, Switzerland; ^3^Department of Psychology, Humboldt-Universität zu Berlin, Berlin, Germany

**Keywords:** Bayesian estimation, Markov chain Monte Carlo, multilevel modeling, structural equation modeling, small sample

## Abstract

Bayesian approaches for estimating multilevel latent variable models can be beneficial in small samples. Prior distributions can be used to overcome small sample problems, for example, when priors that increase the accuracy of estimation are chosen. This article discusses two different but not mutually exclusive approaches for specifying priors. Both approaches aim at stabilizing estimators in such a way that the Mean Squared Error (MSE) of the estimator of the between-group slope will be small. In the first approach, the MSE is decreased by specifying a slightly informative prior for the group-level variance of the predictor variable, whereas in the second approach, the decrease is achieved directly by using a slightly informative prior for the slope. Mathematical and graphical inspections suggest that both approaches can be effective for reducing the MSE in small samples, thus rendering them attractive in these situations. The article also discusses how these approaches can be implemented in M*plus*.

As van de Schoot et al. (2017) pointed out, the number of applications of Bayesian approaches is growing quickly, mainly because software that is easy to use such as M*plus* (Muthén and Muthén, 2012) is providing Bayesian estimation as an option. Bayesian approaches can be beneficial in several respects, for example, by offering greater flexibility (e.g., Hamaker and Klugkist, 2011; Muthén and Asparouhov, 2012; Lüdtke et al., 2013) or fewer estimation problems (e.g., Hox et al., 2012; Depaoli and Clifton, 2015; Zitzmann et al., 2016), particularly when latent variable models are estimated. One major difference between Bayesian and traditional Maximum Likelihood (ML) estimation is that the former not only uses the information from the data at hand (i.e., the likelihood function) but combines it with additional information from what is called the prior distribution. Inferences are based on the result of this combination, that is, the posterior distribution. Scholars have advised researchers against the use of default priors in an automatic fashion and have encouraged them to specify priors on their own (e.g., McNeish, 2016; Smid et al., 2020). This may be an obstacle to some researchers. However, the prior can also be considered a feature of Bayesian estimation that can be used to improve estimation by choosing a favorable prior—a task that is particularly challenging but also particularly worth pursuing when the sample size is small.

The choice of prior has received a lot of attention in the methodological literature (e.g., Natarajan and Kass, 2000; Gelman, 2006; Chung et al., 2013), and scholars have made different suggestions about how priors can be specified in advantageous ways. Only recently, Smid et al. (2020) discussed how priors can be “thoughtfully” constructed on the basis of previous knowledge about the parameter of interest (e.g., on the basis of a previous study or a meta-analysis) in order to reduce small-sample bias. However, it has been argued that the variability of an estimator should not be ignored when evaluating the quality of a method (e.g., Greenland, 2000; Zitzmann et al., 2020), particularly when the sample size is small. Therefore, other suggestions for specifying the prior have been aimed at reducing the Mean Squared Error (MSE), which combines bias and variability: MSE = bias^2^ + variability. One such approach was proposed by Zitzmann et al. (2015), who focused on the between-group slope in multilevel latent variable modeling. The authors suggested that researchers should suitably modify the estimator of the group-level variance of the predictor variable because this will result in a more stable (i.e., more accurate) estimator of the slope. To this end, a slightly informative prior is specified for the group-level variance of the predictor to pull the variance estimates away from zero (i.e., the indirect approach). By doing so, the estimates of the slope will not be too large, and the MSE of the estimator of the slope will be reduced. Notably, in contrast to Smid et al.'s (2020) suggestion, the prior does not need to match previous knowledge or the true value of the parameter in the population. Rather, an incorrect prior whose location deviates from the parameter in the population might reduce the MSE even more than a correct prior will. Zitzmann et al. (2015) found that for a standardized predictor (standardized at Level 1), a slightly informative inverse gamma prior for the group-level variance provided a somewhat biased but much more accurate (because it had a smaller MSE) estimator in small samples. Alternatively, to reduce the MSE of the estimator of the slope, one can specify a slightly informative prior directly for the slope in order to shrink the estimates and thereby ensure that they will not be too large (i.e., the direct approach).

In the present article, we mathematically work out the idea behind the direct approach for a simple multilevel latent variable model, and we contrast this approach with the indirect approach and with ML. Then, we graphically show the benefits that both approaches have over ML when the sample size is small. Finally, we discuss how these approaches can be implemented in M*plus*.

## 1. Example Model

Before we go into detail, we present an example model that we will use later to illustrate the different strategies. The model was suggested by Lüdtke et al. (2008) as one way to yield (asymptotically) unbiased estimates of between-group slopes in contextual studies (see also Asparouhov and Muthén, 2019). To this end, on the group level in the model, the dependent variable *Y* is predicted by a latent variable (i.e., the latent group mean) instead of the unreliable manifest group mean of the predictor variable, which is why the model was named the *multilevel latent covariate model* (Lüdtke et al., 2008). Such latent group means have become part of many more complex multilevel structural equation models that are commonly applied in research practice (see Preacher et al., 2010, 2016, for overviews of such models).

More specifically, the individual-level predictor *X* splits into two uncorrelated and normally distributed parts: a between-group part *X*_*b*_, which is the latent group mean, and a within-group part *X*_*w*_, which is the individual deviation from *X*_*b*_. For a person *i* = 1, …, *n* in group *j* = 1, …, *J*, the decomposition thus reads:

(1) $$\begin{array}{l} X_{ij}=X_{b,j}+X_{w,ij} \end{array}$$

*X*_*b,j*_ is distributed around μ_*X*_ with variance $\tau_{X}^{2}$, whereas the deviation *X*_*w,ij*_ has variance $\sigma_{X}^{2}$. Hereafter, we will also call $\sigma_{X}^{2}$ and $\tau_{X}^{2}$ the within-group and between-group variances of *X*, respectively.

Applying Raudenbush and Bryk's (2002) notation, the regression at the individual level reads:

(2) $$\begin{array}{l} \begin{array}{cc} \text{Level 1:} & Y_{ij}=\beta_{0j}+\beta_{w}X_{w,ij}+\varepsilon_{ij} \end{array} \end{array}$$

where β_*w*_ is the (fixed) within-group slope that describes the relationship between the predictor and the dependent variable at the individual level, and the ε_*ij*_ are normally distributed residuals. The residual variance is $\sigma_{Y}^{2}$. At the group level, the intercept β_0*j*_ is regressed on *X*_*b*_:

(3) $$\begin{array}{l} \begin{array}{cc} \text{Level 2:} & \beta_{0j}=\alpha +\beta_{b}X_{b,j}+\delta_{j} \end{array} \end{array}$$

where α is the overall intercept, and β_*b*_ is the between-group slope (i.e., the relationship between *X* and *Y* at the group level). The δ_*j*_ are normally distributed residuals with variance $\tau_{Y}^{2}$.

Here, we focus on the between-group slope (β_*b*_), which is of great interest in many applications of multilevel models (e.g., in the analysis of contextual effects). When the data are balanced (i.e., equal numbers of persons per group), the ML estimator of β_*b*_ is given by:

(4) $$\begin{array}{l} {\hat{\beta }}_{b}=\frac{{\hat{\tau }}_{YX}}{{\hat{\tau }}_{X}^{2}} \end{array}$$

where ${\hat{\tau }}_{X}^{2}$ and ${\hat{\tau }}_{YX}$ are sample estimators of the group-level variance of *X* and the group-level covariance of *X* and *Y*, respectively.

Some statistical properties of the ML estimator in Equation 4 need to be discussed first to be able to compare this estimator with the Bayesian estimators later on. First, by using the first-order Taylor expansion (e.g., Casella and Berger, 2001; see also Grilli and Rampichini, 2011) and ignoring terms involving higher order factors such as $\frac{1}{n^{2}\left(n-1\right)}$ or $\frac{1}{n^{2}}$ for better readability, then the bias of ${\hat{\beta }}_{b}$ can roughly be approximated by:

(5) $$\begin{array}{l} \text{E}\left({\hat{\beta }}_{b}\right)-\beta_{b}\approx -\frac{2}{J-1}\left\{-\frac{2\left(1-\rho_{X}\right)}{n\rho_{X}}+\frac{1-\rho_{X}}{n\rho_{X}}\left(1+\frac{\beta_{w}}{\beta_{b}}\right)\right\}\beta_{b} \end{array}$$

where $\rho_{X}=\frac{\tau_{X}^{2}}{\tau_{X}^{2}+\sigma_{X}^{2}}$ is the intraclass correlation (ICC) of *X*.^1^ This equation could be simplified further, but we continue to use this expression here to emphasize formal similarities with the biases of the Bayesian estimators (see below). However, even in its current form, it is evident from Equation (5) that the bias critically depends on the sample size (because *J* occurs in the denominator) and that the bias is generally non-zero in small samples. However, if we let *J* become large, the bias diminishes because $\frac{1}{J-1}$ becomes very small—a property of the estimator that we refer to as “asymptotic unbiasedness.” In a similar way, we can yield an approximation of the variability of ${\tilde{\beta }}_{b}$:

(6) $$\begin{array}{l} \text{Var}\left({\hat{\beta }}_{b}\right)\approx \frac{1}{J-1}\{\left[\frac{\rho_{Y}}{\rho_{X}}+\frac{1-\rho_{X}}{n\rho_{X}}\left(\frac{\rho_{Y}}{\rho_{X}}+\frac{1-\rho_{Y}}{1-\rho_{X}}\right)\right]\frac{\tau_{Y}^{2}+\sigma_{Y}^{2}}{\tau_{X}^{2}+\sigma_{X}^{2}}\\ \;+\left[-1-\frac{2\left(1-\rho_{X}\right)}{n\rho_{X}}\frac{\beta_{w}}{\beta_{b}}\right]\beta_{b}^{2}\} \end{array}$$

where $\rho_{Y}=\frac{\tau_{Y}^{2}}{\tau_{Y}^{2}+\sigma_{Y}^{2}}$ is the ICC of *Y*. Similar to the bias, the variability depends on the sample size in such a way that the variability will be small when *J* is large. Because the MSE of ${\hat{\beta }}_{b}$ is the sum of the squared bias and the variability,

(7) $$\begin{array}{l} \text{MSE}\left({\hat{\beta }}_{b}\right)\approx {\left[\text{E}\left({\hat{\beta }}_{b}\right)-\beta_{b}\right]}^{2}+\text{Var}\left({\hat{\beta }}_{b}\right) \end{array}$$

this measure will be small as well. Taken together, the more information the data provide, the more the overall accuracy of the estimator improves.

Whereas the asymptotic properties are favorable, the ML estimator tends to be biased in small samples, and it has high variability and thus a large MSE in these situations (e.g., McNeish, 2017). This challenges the usefulness of the ML estimator when the sample size is small because the result from a single study might be highly inaccurate. Therefore, scholars have called for alternative estimators that are less variable and thus more accurate (i.e., they have a smaller MSE), although they might be more biased than ML. In the multilevel literature, such estimators have been suggested by Chung et al. (2013), Greenland (2000), Grilli and Rampichini (2011), and Zitzmann et al. (2015), for example. Next, we develop the direct strategy, and recap the indirect strategy of specifying the prior.

## 2. The Direct Strategy

We refer to the first strategy as the *direct strategy* because the prior is specified directly for the between-group slope (β_*b*_). To illustrate, we assume a normal prior, which can be formalized as:

(8) $$\begin{array}{l} \beta_{b}\sim \text{N}\left(a,b\right) \end{array}$$

which should be read as “β_*b*_ is normally distributed with mean *a* and variance *b*.” However, for better interpretability, we employ another, more convenient parameterization. Instead of *a* and *b*, we use the terms β_0_ and $\frac{\tau_{Y}^{2}}{\nu_{0}\tau_{X}^{2}}$:

(9) $$\begin{array}{l} \beta_{b}\sim \text{N}\left(\beta_{0},\frac{\tau_{Y}^{2}}{\nu_{0}\tau_{X}^{2}}\right) \end{array}$$

As we will show, β_0_ and ν_0_ can be meaningfully interpreted.

One way of expressing the likelihood for the slope is:

(10) $$\begin{array}{l} \beta_{b}\sim \text{N}\left({\hat{\beta }}_{b},\frac{{\hat{\tau }}_{Y}^{2}}{J{\hat{\tau }}_{X}^{2}}\right) \end{array}$$

where ${\hat{\tau }}_{Y}^{2}$ and ${\hat{\tau }}_{X}^{2}$ are the sampling variances of $\tau_{Y}^{2}$ and $\tau_{X}^{2}$, respectively. If we combine the prior in Equation (9) with the likelihood, we obtain the following posterior:

(11) $$\begin{array}{l} \beta_{b}\sim \text{N}\left(\frac{\nu_{0}}{\nu_{0}+J}\beta_{0}+\frac{J}{\nu_{0}+J}{\hat{\beta }}_{b},\frac{J}{\nu_{0}+J}\frac{{\hat{\tau }}_{Y}^{2}}{J{\hat{\tau }}_{X}^{2}}\right) \end{array}$$

which is also a normal distribution. The mean of this distribution defines the Bayesian Expected A Posteriori (EAP) estimator, which is the standard choice for a point estimator in Bayesian estimation (Note that the Bayes module in M*plus* uses the median of the posterior). With $w=\frac{J}{\nu_{0}+J}$, this Bayesian estimator can also be expressed as:

(12) $$\begin{array}{l} {\bar{\beta }}_{b}=\left(1-w\right)\beta_{0}+w{\hat{\beta }}_{b} \end{array}$$

As can be seen from the equation, the estimator is simply the weighted average of the mean of the prior (β_0_) and ${\hat{\beta }}_{b}$, which suggests straightforward interpretations for the parameters of the prior. One may think of β_0_ as the *prior guess* for the between-group slope and ν_0_ as the *prior sample size* (see also Hoff, 2009). These interpretations are substantiated by the observation that the larger ν_0_, the smaller *w*, and the more the estimates shrink toward β_0_. Less technically speaking, when we are more confident in β_0_, the prior will gain more weight, and the posterior will shift to the mean of the prior. However, when we choose ν_0_ to be very small, *w* will be close to 1, and ${\bar{\beta }}_{b}$ will be similar to ${\hat{\beta }}_{b}$, which justifies the view that the modified estimator includes the original ML estimator as a limiting case. Notice that the prior guess does not need to represent previous knowledge about β_*b*_. Rather, it could be set to a value that is much smaller than what previous studies have suggested and also much smaller than the parameter in the population. However, such an “incorrect” prior guess might still be beneficial, particularly when the sample size is small.

To be able to compare the properties of the Bayesian estimator with the ML estimator and with the Bayesian estimator from the second strategy of specifying the prior, we again use the Taylor expansion, and we ignore terms involving higher order factors. A rough approximation of the bias of ${\bar{\beta }}_{b}$ is then given by:

(13) $$\begin{array}{l} \text{E}\left({\bar{\beta }}_{b}\right)-\beta_{b}\approx \left(1-w\right)\beta_{0}\\ \;+\{-\left(1-w\right)-\frac{2w}{J-1}[-\frac{2\left(1-\rho_{X}\right)}{n\rho_{X}}\\ \;+\frac{1-\rho_{X}}{n\rho_{X}}\left(1+\frac{\beta_{w}}{\beta_{b}}\right)]\}\beta_{b} \end{array}$$

Similar to the ML estimator, ${\bar{\beta }}_{b}$ is generally biased when the sample size is small. However, the bias vanishes when *J* approaches infinity because *w* approaches 1, and $\frac{1}{J-1}$ approaches 0 (asymptotic unbiasedness). Moreover, if ν_0_ is set to a value close to 0, the bias will become similar to the bias of ${\hat{\beta }}_{b}$.

The variability of ${\bar{\beta }}_{b}$ can be approximated as:

(14) $$\begin{array}{l} \text{Var}\left({\bar{\beta }}_{b}\right)\approx \frac{w^{2}}{J-1}\{\left[\frac{\rho_{Y}}{\rho_{X}}+\frac{1-\rho_{X}}{n\rho_{X}}\left(\frac{\rho_{Y}}{\rho_{X}}+\frac{1-\rho_{Y}}{1-\rho_{X}}\right)\right]\frac{\tau_{Y}^{2}+\sigma_{Y}^{2}}{\tau_{X}^{2}+\sigma_{X}^{2}}\\ \;+\left[-1-\frac{2\left(1-\rho_{X}\right)}{n\rho_{X}}\frac{\beta_{w}}{\beta_{b}}\right]\beta_{b}^{2}\} \end{array}$$

With a very large *J*, the variability becomes negligibly small, and the same holds for the MSE. However, the more interesting questions are: How does the MSE of ${\bar{\beta }}_{b}$ depend on the prior parameters (β_0_, ν_0_) when the sample size is small, and how must they be chosen such that the MSE will be smaller than the MSE of ML? Before we compare the different choices for (β_0_, ν_0_), we present another strategy for specifying the prior. Alternatively to specifying the prior directly for the between-group slope, one can also specify a prior for the group-level variance of the predictor, thereby also modifying the estimator of the slope. We call this strategy the *indirect strategy*.

## 3. The Indirect Strategy

The principle that underlies the indirect strategy was discovered in the early years of Structural Equation Modeling (SEM), where models were fit on the basis of the variances and covariances of variables. One observation was that when the sample size was small, covariance matrices tended to be on the border of positive definiteness (e.g., a variance estimate close to 0, correlations close to −1 or 1; e.g., van Driel, 1978; Dijkstra, 1992; Kolenikov and Bollen, 2012). Hence, estimators of slope parameters tended to have high variability and thus also a large MSE. This led Yuan and Chan (2008) to develop the ridge technique to mitigate such problems as it modifies the estimator of the covariance matrix by adding a small value to the main diagonal (see also Yuan and Chan, 2016; Yang and Yuan, 2019). The main idea behind this technique can also be adapted for Bayesian estimation. Papers by Chung et al. (2013), Chung et al. (2015), or Zitzmann et al. (2015) are good examples of this. By means of simulation, Zitzmann et al. (2015) verified that specifying a slightly informative prior for the group-level variance of the predictor that pulls estimates of this variance slightly away from zero can increase the accuracy of the estimator of the between-group slope by reducing its MSE. Note that pulling the variance estimates away from zero corresponds to adding a value to these estimates. A formal argument for why such a prior reduces the MSE was only recently presented by Zitzmann et al. (2020). For reasons of completeness and comparability with the two previously presented estimators, we illustrate the strategy here once more, using the example model from above.

Rather than beginning with the assumption of a normal prior for the between-group slope, we begin with a gamma prior for the inverse of the group-level variance of the predictor variable ($\tau_{X}^{2}$):

(15) $$\begin{array}{l} \frac{1}{\tau_{X}^{2}}\sim \text{Gamma}\left(a,b\right) \end{array}$$

where *a* and *b* are the parameters of the gamma distribution.^2^ Equation (15) reads “$\tau_{X}^{2}$ is inverse-gamma distributed.” As for the normal prior in the previous section, we employ a reparameterization for better interpretability later on. If we set *a* to $\frac{\nu_{0}}{2}$ and *b* to $\frac{\nu_{0}\tau_{0}^{2}}{2}$, the prior reads:

(16) $$\begin{array}{l} \frac{1}{\tau_{X}^{2}}\sim \text{Gamma}\left(\frac{\nu_{0}}{2},\frac{\nu_{0}\tau_{0}^{2}}{2}\right) \end{array}$$

where, as we will show, $\tau_{0}^{2}$ and ν_0_ have interpretations similar to those of the parameters of the (reparameterized) normal prior.

The likelihood for the inverse of the group-level variance can be written as:

(17) $$\begin{array}{l} \frac{1}{\tau_{X}^{2}}\sim \text{Gamma}\left(\frac{J}{2},\frac{J{\hat{\tau }}_{X}^{2}}{2}\right) \end{array}$$

where ${\hat{\tau }}_{X}^{2}$ is the sample variance. Combined with the prior in Equation (16), we yield the inverse gamma posterior:

(18) $$\begin{array}{l} \frac{1}{\tau_{X}^{2}}\sim \text{Gamma}\left(\frac{\nu_{0}+J}{2},\frac{\nu_{0}\tau_{0}^{2}+J{\hat{\tau }}_{X}^{2}}{2}\right) \end{array}$$

As Zitzmann et al. (2020) showed in their Appendix C, the mean of this distribution can be approximated as:

(19) $$\begin{array}{l} {\bar{\tau }}_{X}^{2}\approx \left(1-w\right)\tau_{0}^{2}+w{\hat{\tau }}_{X}^{2} \end{array}$$

where $w=\frac{J}{\nu_{0}+J}$. This equation defines the Bayesian EAP estimator of $\tau_{X}^{2}$. It is interesting to note that the equation resembles Equation (12). The right-hand side of the equation is also a weighted average, and $\tau_{0}^{2}$ and ν_0_ can be thought of as the prior guess and the prior sample size, respectively (see Hoff, 2009; Lüdtke et al., 2018; Zitzmann et al., 2020).

Adding a prior for $\tau_{X}^{2}$ also has consequences for the estimator of the between-group slope (β_*b*_). Replacing the denominator in Equation (4) (${\hat{\tau }}_{X}^{2}$) with ${\bar{\tau }}_{X}^{2}$ results in:

(20) $$\begin{array}{l} {\tilde{\beta }}_{b}=\frac{{\hat{\tau }}_{YX}}{\left(1-w\right)\tau_{0}^{2}+w{\hat{\tau }}_{X}^{2}} \end{array}$$

This new estimator is indicated by a tilde (~) in order to better differentiate it from the ML estimator and from the Bayesian estimator that results from the direct strategy of specifying the prior (Equation 12).

To derive some properties of ${\tilde{\beta }}_{b}$, we apply exactly the same reasoning that led to the respective properties of ${\hat{\beta }}_{b}$ and ${\bar{\beta }}_{b}$. Accordingly, the bias of ${\tilde{\beta }}_{b}$ is roughly:

(21) $$\begin{array}{l} \text{E}\left({\tilde{\beta }}_{b}\right)-\beta_{b}\approx \left(f-1\right)\beta_{b}-\frac{2wf^{2}}{J-1}\{-wf\left[1+\frac{2\left(1-\rho_{X}\right)}{n\rho_{X}}\right]+1\\ \;+\frac{1-\rho_{X}}{n\rho_{X}}\left(1+\frac{\beta_{w}}{\beta_{b}}\right)\}\beta_{b} \end{array}$$

where *f* is used as an abbreviation for the ratio $\frac{\tau_{X}^{2}}{\left(1-w\right)\tau_{0}^{2}+w\tau_{X}^{2}}$.^3^ Notice that the equation implies that ${\tilde{\beta }}_{b}$ is generally biased when the sample size is finite, whereas the bias diminishes when *J* approaches infinity (asymptotic unbiasedness). Moreover, the bias becomes similar to the biases of ${\bar{\beta }}_{b}$ and ${\hat{\beta }}_{b}$ when we let ν_0_ become very small.

The variability of ${\tilde{\beta }}_{b}$ is:

(22) $$\begin{array}{l} \text{Var}\left({\tilde{\beta }}_{b}\right)\approx \frac{f^{2}}{J-1}\{\left[\frac{\rho_{Y}}{\rho_{X}}+\frac{1-\rho_{X}}{n\rho_{X}}\left(\frac{\rho_{Y}}{\rho_{X}}+\frac{1-\rho_{Y}}{1-\rho_{X}}\right)\right]\frac{\tau_{Y}^{2}+\sigma_{Y}^{2}}{\tau_{X}^{2}+\sigma_{X}^{2}}\\ \;+[2wf(wf\left(1+\frac{2\left(1-\rho_{X}\right)}{n\rho_{X}}\right)\\ \;-2\left(1+\frac{1-\rho_{X}}{n\rho_{X}}\left(1+\frac{\beta_{w}}{\beta_{b}}\right)\right))+1\\ \;+\frac{2\left(1-\rho_{X}\right)}{n\rho_{X}}\frac{\beta_{w}}{\beta_{b}}]\beta_{b}^{2}\} \end{array}$$

Similar to the previous equation, we can easily infer that when *J* is large, the variability will be small and, thus, the MSE, which combines bias and variability, will be small as well—an observation that once again demonstrates the role of the sample size in determining the accuracy of estimations. However, as mentioned above, it is much more interesting to ask how the prior parameters $\tau_{0}^{2}$ and ν_0_ must be chosen such that the MSE will be reduced in comparison with ML in small samples.

## 4. Comparing the MSEs in Small Samples

In this section, we investigate the MSE of the different strategies for specifying priors in small samples for different choices of the prior parameters, using the example model from above to simulate data that are typical in psychology. Because it is difficult to infer from the equations how the MSEs compare with each other, they were plotted against the sample size to allow for graphical comparisons.

In accordance with Lüdtke et al. (2008), we considered the case of standardized variables (standardized at Level 1), and we assumed that the between-group slope (β_*b*_) was 0.7 in the population. Moreover, we set the number of persons per group (*n*) to 5, which is not uncommon in many subdisciplines of psychology, including organizational, personality, and social psychology. The ICC of the predictor was 0.1, which could be considered small- to medium-sized compared with typical ICCs (Snijders and Bosker, 2012; Zitzmann et al., 2015). The sample size at the group level (*J*) was varied from 20 to 60 groups because these numbers represent small sample sizes (e.g., Hox et al., 2012; see also Hox et al., 2010) and the aim was to compare the estimators in these situations.

**Figure 1:**
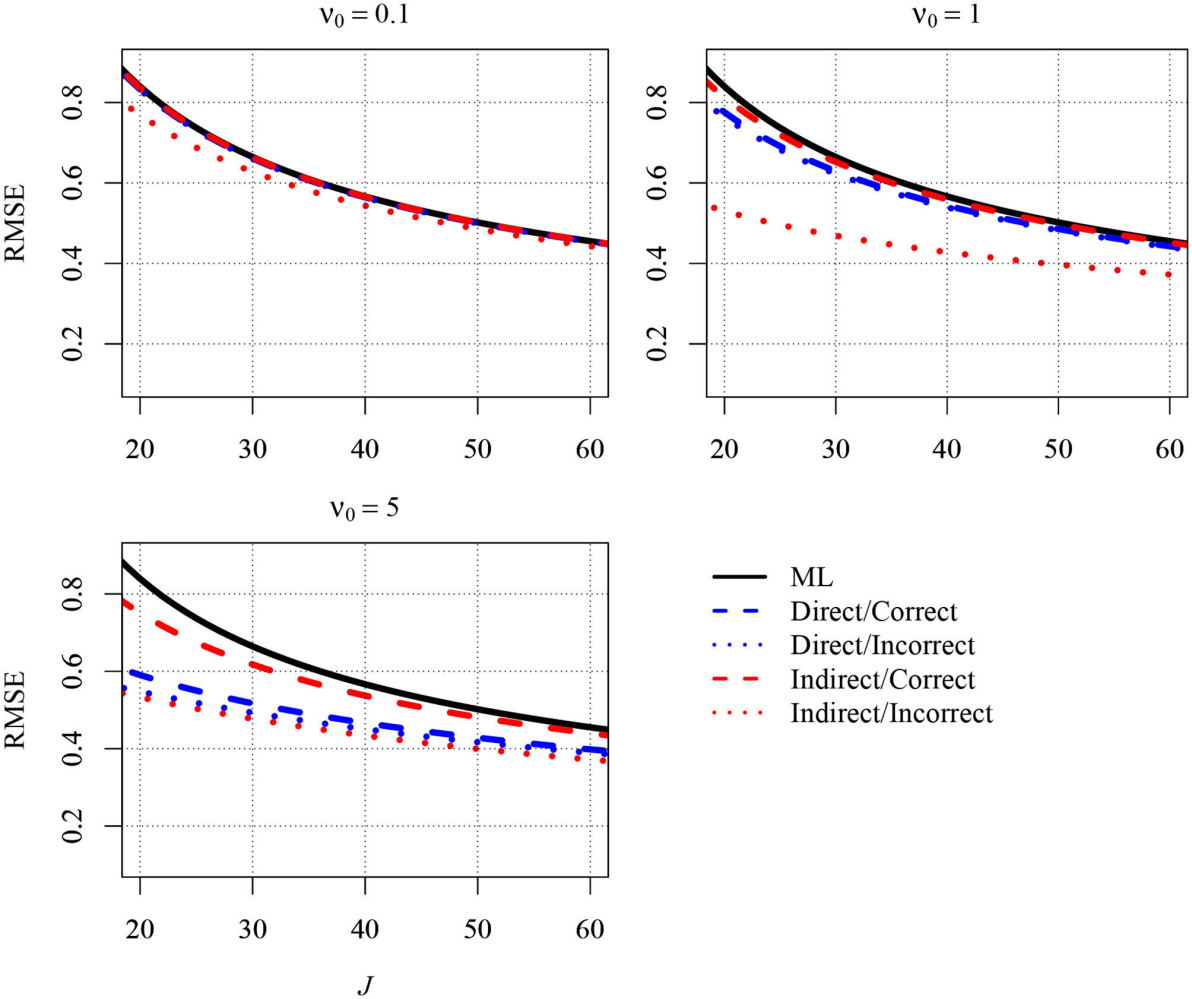
The analytically derived Root Mean Squared Error (RMSE) in estimating the between-group slope for the direct and the indirect approach as a function of the sample size at the group level (*J*) and the prior distribution. Results are shown for *n* = 5 persons per group and an intraclass correlation of ICC = 0.1. ML, maximum likelihood; direct, the prior was specified directly for the between-group slope; indirect, the prior was specified for the group-level variance of the predictor variable; correct, correct prior (i.e., the prior guess equaled the value of the parameter in the population); incorrect, incorrect prior (i.e., the prior guess deviated from the parameter in the population); ν_0_, prior sample size.

Figure 1 depicts a normalized version of the MSE, the Root Mean Squared Error (RMSE), for five different estimators of the slope. The first estimator in the figure is the ML estimator (solid black line). The second estimator (blue dashed line) is the Bayesian estimator that results when the direct strategy is combined with a correct prior for β_*b*_ (i.e., the prior guess, β_0_, equals the parameter in the population). Because β_*b*_ was 0.7 in the population, a correct prior for β_*b*_ was specified by setting β_0_ equal to this value. The third estimator (blue dotted line) also resulted from the direct strategy. However, β_0_ was set to 0 (and thus well below 0.7) in order to shrink estimates that were too large toward zero. The fourth estimator (red dashed line) resulted from the indirect strategy with a correct prior for the group-level variance of the predictor ($\tau_{X}^{2}$). The prior guess ($\tau_{0}^{2}$) was set to 0.1, which was the value of $\tau_{X}^{2}$ in the population.^4^ The fifth estimator (red dotted line) resulted from the indirect strategy as well. However, β_0_ was set to 1, which was above the parameter in the population. Thus, estimates of the variance were pulled away from zero, and, therefore, the estimates of the slope were shrunken. The three different panels of Figure 1 show the RMSEs for different values of ν_0_: 0.1 (upper left), 1.0 (upper right), and 5.0 (lower left). The first two values can be considered choices that are only slightly informative, whereas the latter is more informative and was used here to illustrate what happens to the RMSE when the priors become more informative.

**Figure A1:**
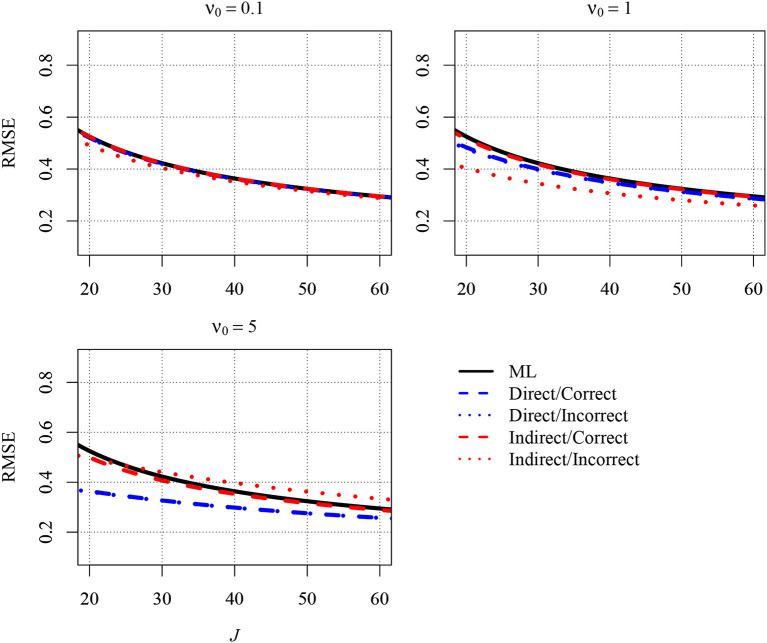
The analytically derived Root Mean Squared Error (RMSE) in estimating the between-group slope for the direct and the indirect approach as a function of the sample size at the group level (*J*) and the prior distribution. Results are shown for *n* = 10 persons per group and an intraclass correlation of ICC = 0.1. ML, maximum likelihood; direct, the prior was specified directly for the between-group slope; indirect, the prior was specified for the group-level variance of the predictor variable; correct, correct prior (i.e., the prior guess equaled the value of the parameter in the population); incorrect, incorrect prior (i.e., the prior guess deviated from the parameter in the population); ν_0_, prior sample size.

**Figure A2:**
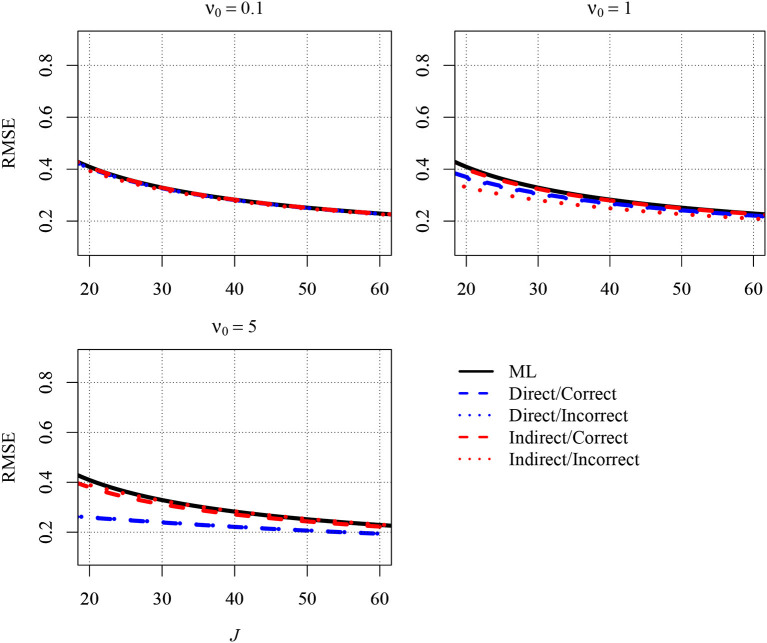
The analytically derived Root Mean Squared Error (RMSE) in estimating the between-group slope for the direct and the indirect approach as a function of the sample size at the group level (*J*) and the prior distribution. Results are shown for a small number of *n* = 5 persons per group and an intraclass correlation of ICC = 0.2. ML, maximum likelihood; direct, the prior was specified directly for the between-group slope; indirect, the prior was specified for the group-level variance of the predictor variable; correct, correct prior (i.e., the prior guess equaled the value of the parameter in the population); incorrect, incorrect prior (i.e., the prior guess deviated from the parameter in the population); ν_0_, prior sample size.

As can be seen in the Figure 1, the different estimators tended to provide different RMSEs. The RMSE was largest for the ML estimator, whereas the RMSE was reduced when a Bayesian estimator was used. The reduction was particularly pronounced when *J* was very small. In addition and more important, the extent of the reduction also depended on the strategy for specifying the prior and the choices for the prior parameters. Although the direct strategy reduced the RMSE overall, the RMSE was slightly smaller when this strategy was combined with an incorrect prior (i.e., β_0_ = 0) than with a correct prior (i.e., β_0_ = 0.7). Moreover, the choice of a larger ν_0_ was associated with a smaller RMSE. However, the smallest RMSEs emerged when the indirect strategy was used with an incorrect prior (i.e., $\tau_{0}^{2}=1$, which was also the upper bound of $\tau_{X}^{2}$ due to standardization). With a larger value of ν_0_ = 1, the RMSE was reduced relative to a ν_0_ of 0.1. However, setting ν_0_ to 5 did not yield an RMSE that was even smaller. Rather, the RMSE was slightly larger than with a ν_0_ of 1 because the bias induced by the prior outweighed the variability in the computation of the RMSE. Additional results are presented in the Appendix. Figure A1 shows the RMSEs of the different estimators for a larger number of 10 persons per group, whereas Figure A2 shows the RMSEs for a higher ICC of .2. Although the RMSEs were smaller in Figures A1, A2 compared with Figure 1, the big picture was similar overall: The different estimators provided different RMSEs. All Bayesian estimators provided smaller RMSEs than the ML estimator in very small samples except the indirect strategy with an incorrect informative prior.

To sum up, both strategies for specifying the prior offer attractive ways to obtain more accurate estimators of the between-group slope in small samples when used with slightly informative priors. Especially when no previous knowledge exists about the parameters, the choice of a relatively small prior guess for the between-group slope or a relatively large prior guess for the group-level variance of the predictor could be useful when these choices are combined with a small ν_0_ in the low one-digit range. Although somewhat biased, the resulting Bayesian estimators of the slope were found to be more accurate than ML when the sample size was small.

## 5. Discussion

**Figure 2:**
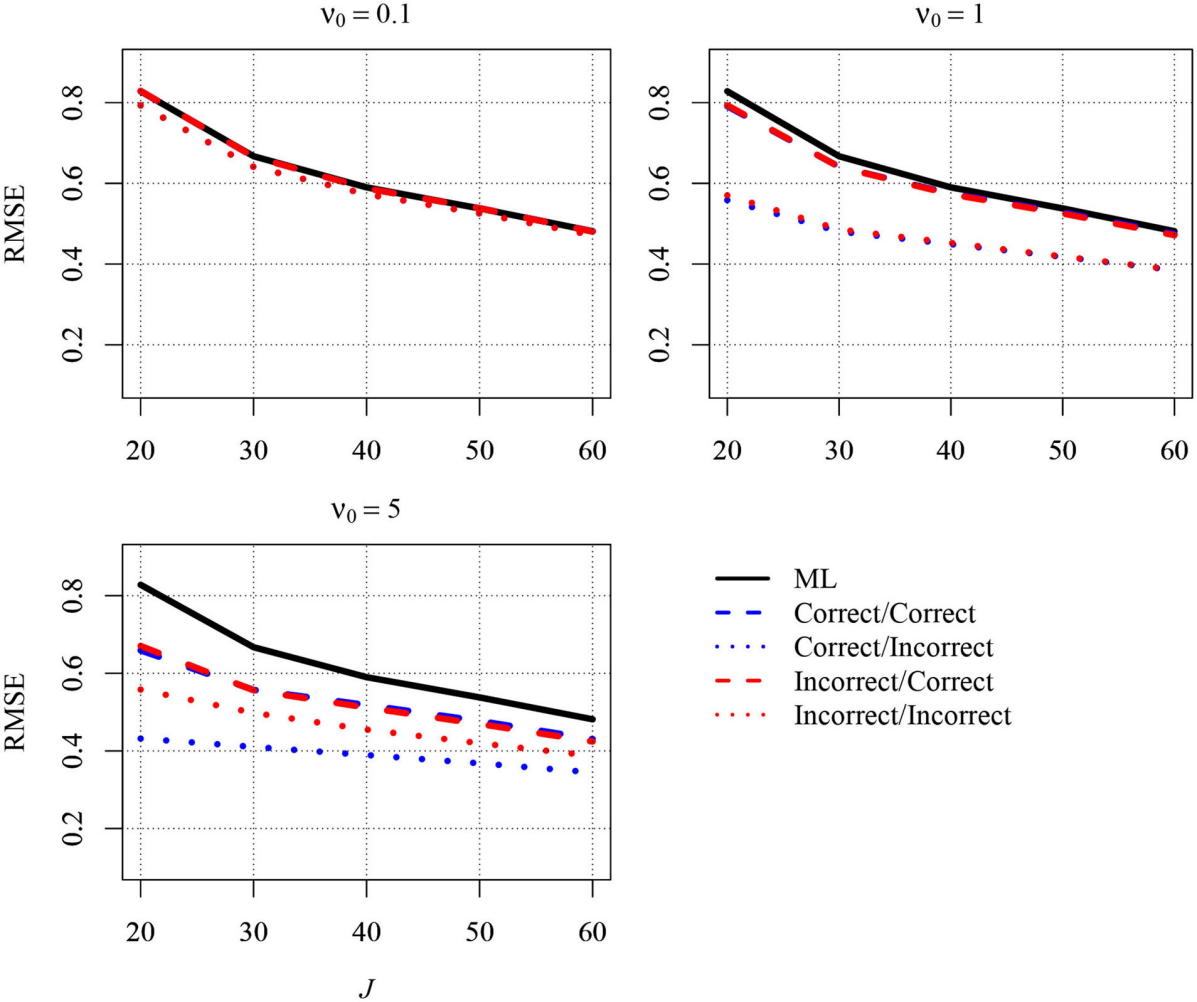
The simulated Root Mean Squared Error (RMSE) in estimating the between-group slope for the combined approach as a function of the sample size at the group level (*J*) and the prior distribution. Results are shown for *n* = 5 persons per group and an intraclass correlation of ICC = 0.1. ML, maximum likelihood; correct/correct, correct priors (i.e., the prior guesses equaled the values of the parameters in the population) were specified for the between-group slope and the group-level variance of the predictor variable; correct/incorrect, a correct prior was specified for the between-group slope, and an incorrect prior (i.e., the prior guess deviated from the parameter in the population) was specified for the group-level variance of the predictor variable; incorrect/correct, an incorrect prior was specified for the between-group slope, and a correct prior was specified for the group-level variance of the predictor variable; incorrect/incorrect, incorrect priors were specified for the between-group slope and the group-level variance of the predictor variable; ν_0_, prior sample size.

It has been argued that Bayesian approaches can be beneficial when the sample size is small because prior distributions can be used to increase estimation accuracy. In the present article, we focused on the between-group slope because this parameter is often of interest in multilevel latent variable modeling. Two approaches for specifying priors can be distinguished, both of which are aimed at reducing the MSE of the estimator of the between-group slope: In the first approach, a slightly informative prior is specified directly for the slope, whereas in the indirect approach, the MSE is reduced by using a slightly informative prior for the group-level variance of the predictor variable. In the present article, we worked out the former approach mathematically and compared it with the indirect approach and with ML. Graphical inspections suggested that both approaches can be very effective in reducing the MSE compared with ML in small samples, rendering them attractive for researchers. We would like to add that these approaches are not mutually exclusive and that researchers can also apply them simultaneously by specifying slightly informative priors for the slope as well as for the group-level variance of the predictor variable. To provide initial information about how such a simultaneous application of the two approaches performs, we conducted an additional simulation study with 20 to 60 groups, 5 persons per group, and an ICC of the predictor variable of 0.1. Figure 2 depicts the RMSE for five different estimators of the slope. The first estimator is the ML estimator (solid black line). The second estimator (blue dashed line) is the Bayesian estimator that resulted when the direct strategy and the indirect strategy were simultaneously applied and combined with correct priors for the between-group slope and the group-level variance of the predictor, respectively. The third estimator (blue dotted line) also resulted from combining the two strategies. However, whereas the direct strategy was combined with a correct prior, the indirect strategy was combined with an incorrect prior. The fourth estimator (red dashed line) resulted from simultaneously applying the direct strategy with an incorrect prior and the indirect strategy with a correct prior. The fifth estimator (red dotted line) resulted from the simultaneous application of the two strategies as well. However, both strategies were combined with incorrect priors. The three different panels of the figure show the RMSEs for different values of the prior sample size. Again, the RMSE was largest for the ML estimator, whereas the RMSE was reduced when a Bayesian estimator was used, particularly when this estimator was combined with slightly informative priors and the sample size was small. Thus, the overall finding from this simulation confirmed that a simultaneous application of the two approaches (i.e., specifying slightly informative priors for the slope as well as for the group-level variance of the predictor variable) can also be beneficial. However, because the consequences of such a use could not be studied exhaustively here, it would be interesting to conduct a more thorough simulation on this topic in future research.

Although our findings were generally favorable and could be considered a successful “proof of concept,” a word of caution is nevertheless needed. Our demonstrations were very limited. For example, the specific conditions we studied do not completely reflect real data. Future research should consider a wider range of conditions for more conclusive findings. Moreover, the example model we used was overly simple. Realistic models typically involve more than one predictor and also multiple indicators per construct. However, one can derive the Bayesian estimators analogously in this more general multivariate case. Zitzmann (2018) even showed that in a multilevel SEM with two latent predictors with three indicators each, a slightly informative inverse Wishart prior for the covariance matrix of the predictors led to more accurate estimators of the between-group slopes, particularly when the samples size was small. Finally, the MSEs of the estimators we derived were only rough approximations. These approximations can nevertheless be useful for deriving hypotheses about which prior works well under which condition.

Before we come to M*plus*, we wish to acknowledge that parameter stabilization does not require Bayesian estimation. In fact, the idea of using slightly informative priors is similar to using techniques from the frequentist framework (Hastie et al., 2009). For example, the weighting parameter (*w*) of the Bayesian estimator in Equation (12) has an effect similar to that achieved by the penalty in regularized SEM (e.g., Jacobucci et al., 2016), and the weighting parameter in Equation (19) corresponds with the tuning parameter in ridge generalized least squares (e.g., Yuan and Chan, 2016) and regularized consistent partial least squares estimation (e.g., Jung and Park, 2018). Despite the existence of these methods, we employed Bayesian estimation here for reasons of convenience and because this type of estimation is an option in M*plus*, which is the software that many researchers use to fit multilevel latent variable models.

M*plus* does not use Bayesian estimation as the default, and users must request it by setting ESTIMATOR to BAYES. Next, to yield a more accurate estimator of the between-group slope by using a slightly informative prior for this parameter, users must specify such a prior manually. In M*plus*, normal priors are parameterized as in Equation (8), where *a* is the mean and *b* is the variance. Thus, to specify a normal prior with the prior guess (β_0_) and the prior sample size (ν_0_) equaling 0 and 1, respectively, users must compute *a* and *b* first. Given *a* = β_0_ and $b=\frac{\tau_{Y}^{2}}{\nu_{0}\tau_{X}^{2}}$, we yield an *a* of 0 and a *b* of $\frac{\tau_{Y}^{2}}{\tau_{X}^{2}}$. Because $\tau_{Y}^{2}$ and $\tau_{X}^{2}$ are unknown, they need to be replaced with, for example, their sample estimates. Assuming that these estimates are ${\hat{\tau }}_{Y}^{2}=0.15$ and ${\hat{\tau }}_{X}^{2}=0.1$, then the prior is specified by the following line of code:


  MODEL PRIORS:
     Name of slope ~ N(0, 1.5);


Our findings suggest that this prior increases the accuracy of estimation in small samples. Choosing an even smaller value for *b* can also be useful in these situations. Alternatively, one could also specify a slightly informative prior for the group-level variance of the predictor. To be able to do this, users must compute the parameters *a* and *b* in Equation (15) because M*plus* uses this parameterization of the inverse gamma prior. Setting both $\tau_{0}^{2}$ and ν_0_ to 1 results in $a=b=\frac{1}{2}$, using $a=\frac{\nu_{0}}{2}$ and $b=\frac{\nu_{0}\tau_{0}^{2}}{2}$. The following code line implements the prior with M*plus*:


  MODEL PRIORS:
     Name of variance ~ IG(0.5, 0.5);


For a standardized predictor, this prior is quite effective when the sample size is small. Specifying somewhat larger values (e.g., by setting ν_0_ = 2) might increase estimation accuracy even further (Depaoli and Clifton, 2015).

To conclude, we worked out and discussed Bayesian approaches that perform better than ML in small samples, and we offered some practical guidance on how to implement these approaches with M*plus*. We hope that this article will help researchers in the field of psychology move beyond using Bayesian estimation as “just another estimator” and will help them make choices that are beneficial when their aim is to fit multilevel latent variable models and the sample size is small.

## Data Availability Statement

The original contributions presented in the study are included in the article/supplementary material, further inquiries can be directed to the corresponding author/s.

## Author Contributions

SZ: writing, mathematical derivations, and graphic design. CH: writing. MH: writing and lead. All authors contributed to the article and approved the submitted version.

## Conflict of Interest

The authors declare that the research was conducted in the absence of any commercial or financial relationships that could be construed as a potential conflict of interest.

## Footnotes

^1^The ICC quantifies the amount of the total variance that can be attributed to differences between the groups (e.g., Snijders and Bosker, 2012).

^2^The inverse of a variance is sometimes also referred to as the precision in the statistical literature (e.g., Hoff, 2009).

^3^We would like to state that we recognized a typo in the bias formula of Zitzmann et al.'s (2020) original publication. There should be a minus sign in front of the first term of the curly-bracketed expression. Equation (21) presents the corrected formula. However, the numerical results on which Figure 3 in Zitzmann et al. (2020) was based were not affected by the typo because these results were generated from formulas that were correct and also provided even more precise approximations than the ones presented in the article (because terms with higher order factors were not omitted).

^4^Because of the standardization, $\tau_{X}^{2}$ is equal to the value of the ICC.
